# An Unusual Presentation of an Epidermoid Cyst Near the Nasolabial Fold: A Case Report

**DOI:** 10.7759/cureus.100019

**Published:** 2025-12-24

**Authors:** Abira Chattopadhyay, Md Arif Hossain, Aritra Chatterjee, Nayana De, Basabdatta Ghosh

**Affiliations:** 1 Oral and Maxillofacial Surgery, Dr R Ahmed Dental College and Hospital, Kolkata, IND

**Keywords:** cheek swelling, epidermoid cyst, head and neck lesion, intra-oral excision, nasolabial fold

## Abstract

Epidermoid cysts are benign, slow-growing lesions arising from epidermal cell proliferation within the dermis. Although common on the scalp and trunk, they rarely occur in the head and neck region, particularly the cheek. We report a 31-year-old male with a painless, progressive swelling lateral to the right nasolabial fold for two years. Ultrasonography and fine-needle aspiration yielded keratinous material suggestive of an epidermoid cyst. The lesion was excised entirely via an intraoral approach under general anesthesia. Histopathology showed a cyst lined by orthokeratinized stratified squamous epithelium without adnexal structures, confirming an epidermoid cyst. Healing was uneventful, and no recurrence was noted after one year. This case highlights the importance of considering epidermoid cysts in the differential diagnosis of cheek masses and demonstrates that an intraoral approach provides excellent aesthetic and functional results.

## Introduction

Epidermoid cysts, also termed epidermal inclusion or infundibular cysts, are benign developmental lesions resulting from the proliferation of epidermal cells within the dermis [1]. They account for approximately seven percent of cysts in the head and neck region, with only about 1.6% presenting intraorally [1-3]. These cysts may be congenital, due to ectodermal entrapment during embryonic fusion, or acquired following trauma or surgery [4,5].

In the oral and maxillofacial region, dermoid and epidermoid cysts typically occur along midline structures such as the floor of the mouth. Localization to areas such as the cheek or the nasolabial fold is considerably less common, likely due to the absence of embryologic fusion planes that predispose to congenital cyst formation [2,5]. Most cases are diagnosed in young or middle-aged adults, and many lesions remain unnoticed until they reach a cosmetically or functionally significant size. The differential diagnosis includes dermoid cyst, lipoma, nasolabial (Klestadt’s) cyst, and schwannoma, which should be excluded based on imaging, cytology, and histology [2,3]. When evaluating cheek swellings, differential diagnoses include dermoid cysts, lipomas, schwannomas, and nasolabial cysts.

In the head and neck region, the submental and submandibular spaces and the floor of the mouth are most commonly affected, whereas cheek or nasolabial presentations are rare [2,5]. Accurate assessment and preservation of facial soft-tissue contours are particularly important in aesthetically sensitive regions such as the nasolabial fold. Advances in three-dimensional imaging have significantly improved the evaluation of facial soft-tissue changes [6,7]. This report presents an uncommon epidermoid cyst near the nasolabial fold, successfully managed via an intraoral approach, emphasizing diagnostic considerations and aesthetic outcomes [1-5].

## Case presentation

**Figure 1:**
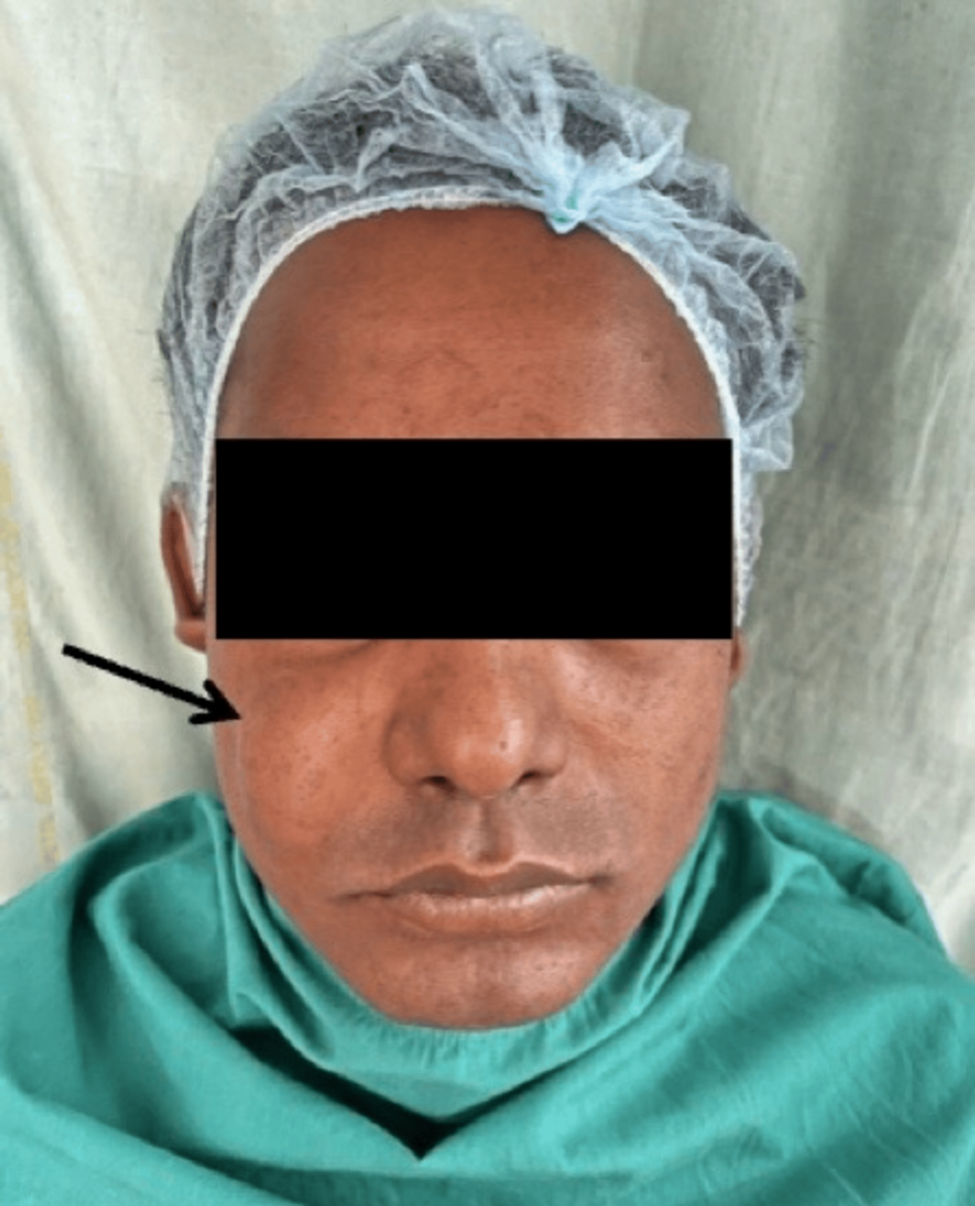
Preoperative clinical photograph. Distinct oval swelling lateral to the right nasolabial fold

A 31-year-old male presented to the Department of Oral and Maxillofacial Surgery with a painless, gradually enlarging swelling on the right cheek near the nasolabial fold, present for two years (Figure 1). There was no history of trauma, infection, or discharge, and the patient reported cosmetic concern and mild discomfort while smiling.

**Figure 2:**
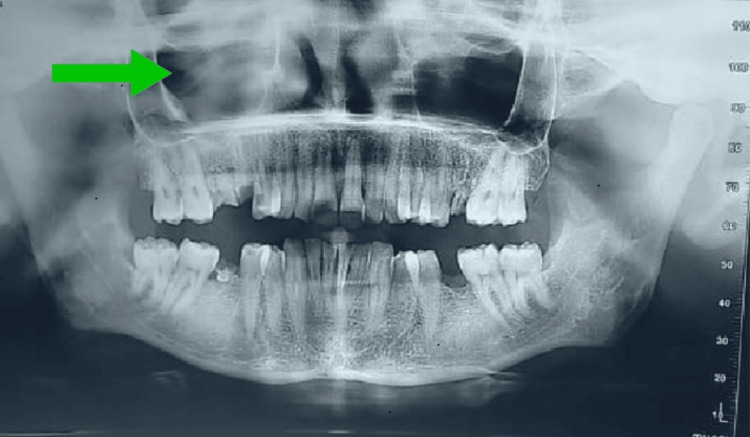
Preoperative orthopantomogram. Orthopantomogram with specifications of 12.70 seconds, 74.00 kV and 6.00 mA showing soft-tissue shadow over the right canine-premolar region without bony changes.

On extraoral examination, the swelling was oval, firm, mobile, and non-tender, measuring approximately 4 × 3 cm. The overlying skin was normal in colour and texture. Intraoral mucosa appeared normal. Orthopantomogram (OPG) revealed no bony involvement (Figure 2). Ultrasonography demonstrated a well-defined, heterogeneously hyperechoic subcutaneous lesion with minimal internal vascularity, and fine-needle aspiration produced thick keratinous material containing anucleate squames-findings consistent with an epidermoid or dermoid cyst.

**Figure 3:**
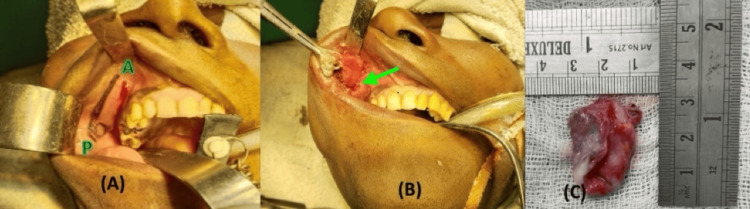
Intraoral approach for cyst excision via the right maxillary buccal sulcus. (A) Placement of incision extending from anterior (A) to posterior (P) region in the right maxillary buccal sulcus. (B) Dissection and enucleation of the cystic lesion through the intraoral approach. (C) Excised cystic specimen measuring approximately 3 × 2 cm.

The intraoral approach was preferred as it allows satisfactory exposure of the lesion while avoiding external scarring, thereby ensuring a better cosmetic outcome. An intraoral vestibular incision was placed under general anesthesia in the right maxillary buccal sulcus below the parotid duct orifice (Figure 3A). Careful, sharp, and blunt dissection permitted enucleation of the cyst in toto (Figure 3B). Accidental puncture released thick, cheese-like keratinous debris; the cavity was irrigated with saline and closed with 3-0 vicryl sutures. The specimen was sent for histopathological examination (Figure 3C).

**Figure 4:**
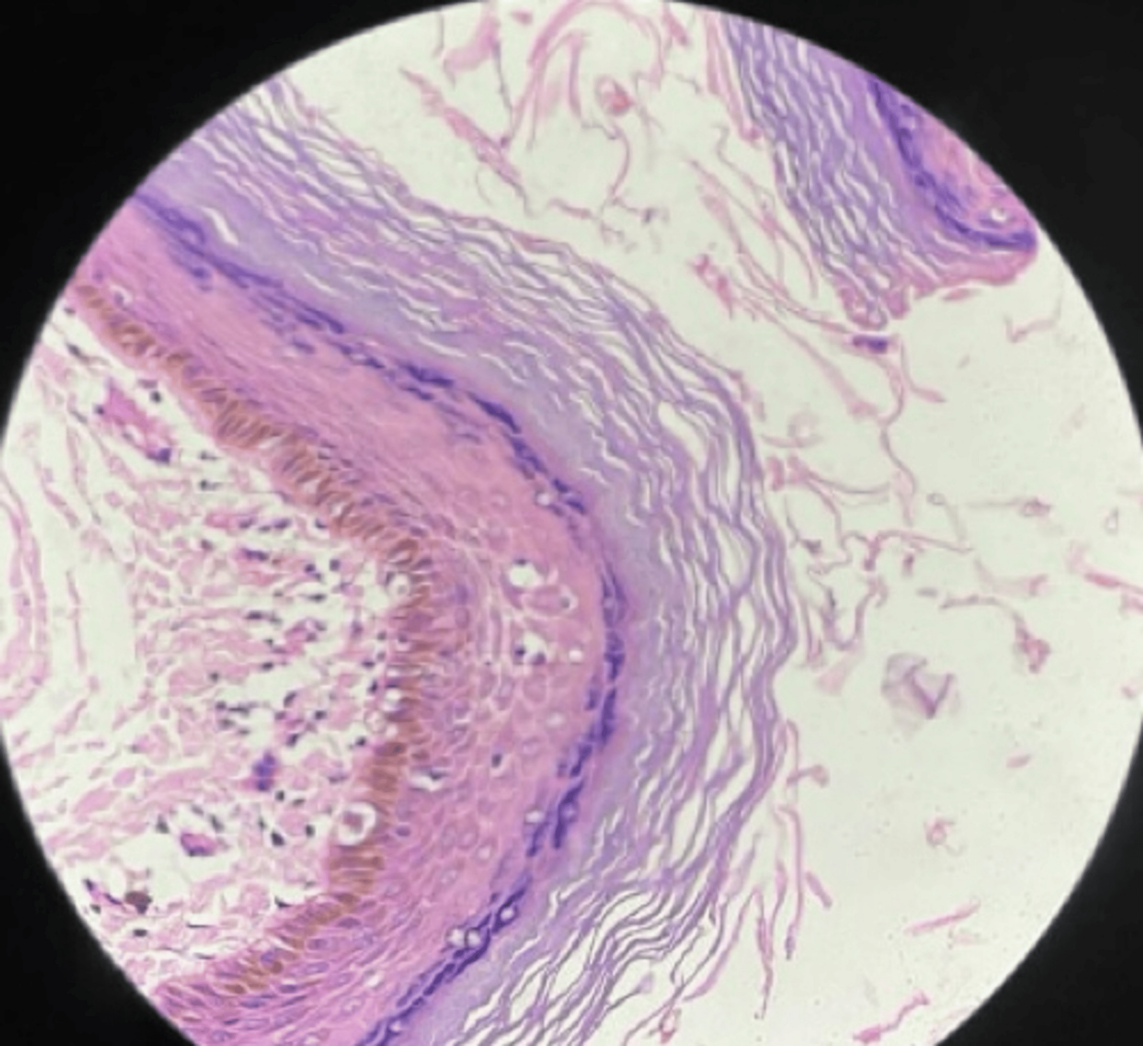
Histopathological findings confirming epidermoid cyst. Photomicrograph showing a cystic cavity lined by orthokeratinized stratified squamous epithelium without adnexal structures and a lumen filled with laminated keratin debris (H&E stain, 40×).

Microscopy revealed a cystic lumen lined by orthokeratinized stratified squamous epithelium lacking adnexal structures, and a lumen filled with laminated keratin debris, confirming an epidermoid cyst (Figure 4).

**Figure 5:**
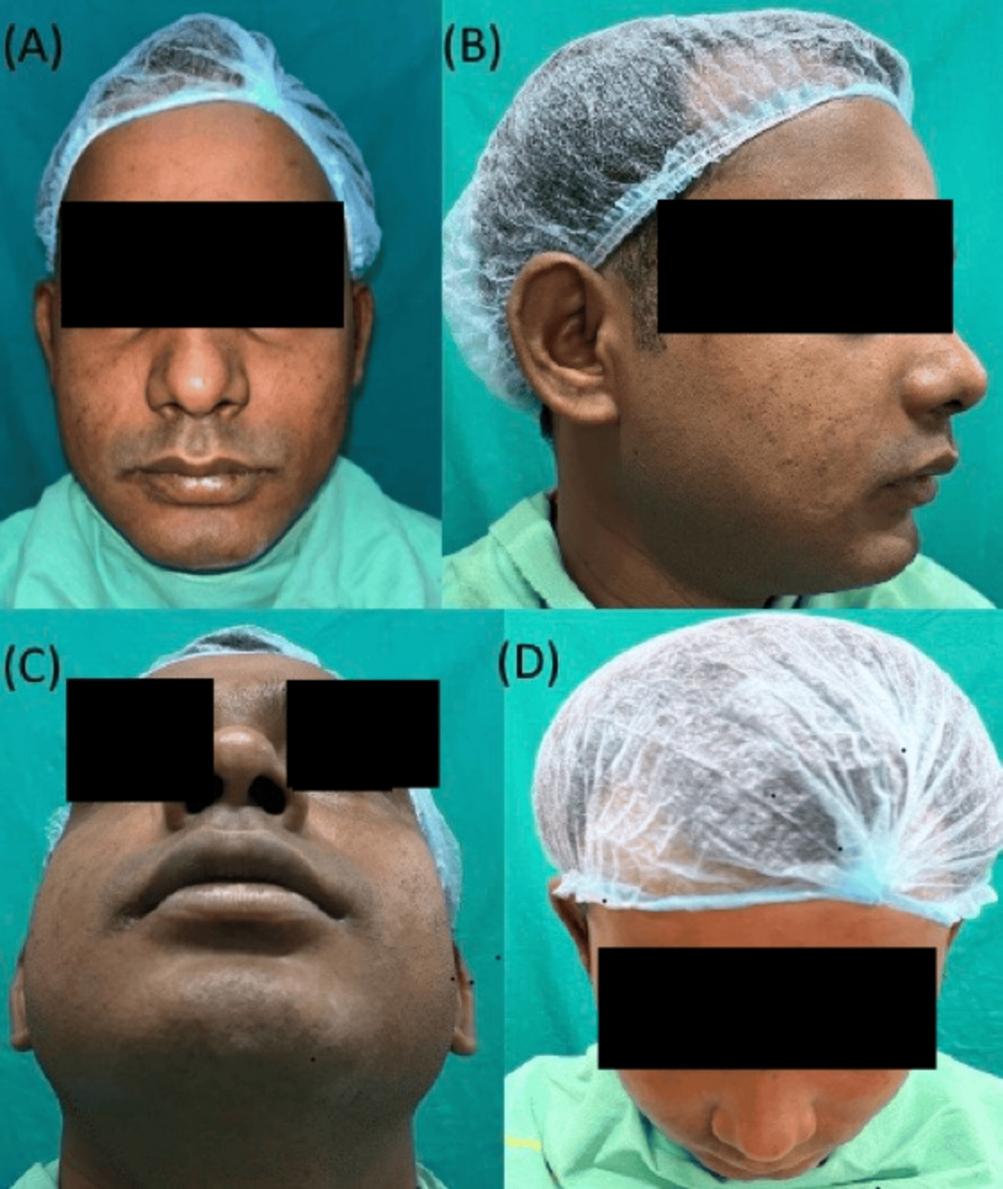
Postoperative images after one year. (A) Frontal, (B) Lateral, (C) Basal, and (D) Superior views showing symmetrical facial contour and complete healing with no visible scar or recurrence one year after intraoral excision of the epidermoid cyst.

Postoperative recovery was uneventful. Because the approach was intraoral, there was no visible facial scar. At one-year follow-up, the patient remained asymptomatic with no recurrence (Figure 5).

## Discussion

Epidermoid cysts account for only a small fraction of cystic lesions in the head and neck region and are infrequently encountered in the cheek or nasolabial area [1-3]. They are histologically classified as epidermoid (lined by squamous epithelium only), dermoid (epithelium plus adnexal structures), or teratoid (elements from multiple germ layers) [5,8].

Their origin may be congenitally caused by ectodermal entrapment during embryogenesis, or acquired via implantation of epidermal elements after trauma or surgery [4,9,10]. Clinically, epidermoid cysts present as slow-growing, well-circumscribed, painless masses with intact overlying skin; the absence of a punctum may render diagnosis challenging. Ultrasonography and fine-needle aspiration cytology are helpful preliminary investigations for superficial lesions, while CT or MRI may be needed for deep or atypical presentations [1]. Definitive diagnosis rests on histopathology [2].

The differential diagnosis includes dermoid cyst, lipoma, nasolabial (Klestadt’s) cyst, and schwannoma, which should be excluded based on imaging, cytology, and histology [2,3]. When evaluating cheek swellings, differential diagnoses include dermoid cysts, lipomas, schwannomas, and nasolabial cysts. Complete surgical excision is the treatment of choice, and recurrence is uncommon when the excision is thorough [11]. While extraoral access is conventional for cheek lesions, the intraoral approach offers cosmetic superiority for nasolabial or midfacial cysts by avoiding visible scars and preserving aesthetics [2,11]. Although benign, epidermoid cysts have been reported to undergo malignant transformation into squamous cell carcinoma, basal cell carcinoma, or Merkel cell carcinoma in rare cases [11]. The incidence of malignant change from an epidermal cyst to cutaneous squamous cell carcinoma is 0.011-0.045% [12]

The cheek is an uncommon location because it does not correspond to a recognized embryologic fusion line, unlike the midline structures of the oral cavity [13]. Nasolabial cysts in particular may resemble epidermoid cysts but usually occur more superficially and contain mucous rather than keratinous material [14]. Recognition of these distinctions is paramount in devising an appropriate treatment plan. 

## Conclusions

Epidermoid cysts of the cheek are rare and can clinically mimic other benign soft-tissue swellings. A systematic clinical assessment, supported by imaging and confirmed by histopathology, ensures accurate diagnosis and management. When anatomically feasible, intraoral excision provides effective, scar-free removal with excellent cosmetic and functional outcomes and minimal recurrence risk. Although this report describes a single case, it highlights an uncommon clinical presentation and underscores the need for larger case series with longer follow-up to better define optimal management and outcomes.
